# Influence of Improved Microclimate Conditions on Growth and Physiological Performance of Two Japanese Quail Lines

**DOI:** 10.3390/ani13061118

**Published:** 2023-03-22

**Authors:** Ahmed M. Emam, Shaaban S. Elnesr, Ensaf A. El-Full, Bothaina Y. Mahmoud, Hamada Elwan

**Affiliations:** 1 Department of Poultry Production, Faculty of Agriculture, Fayoum University, Fayoum 63514, Egypt; 2 Animal and Poultry Production Department, Faculty of Agriculture, Minia University, El-Minya 61519, Egypt

**Keywords:** microclimate, zeolite, selection, physiology, quail

## Abstract

**Simple Summary:**

Ammonia emission in poultry farms leads to environmental or health problems. Additionally, the sustainability and health of poultry depend on the improvement of the surrounding microclimate conditions of birds (ammonia, ambient temperature, heat index, and relative humidity). Therefore, the objective of this study was to investigate the influence of the addition of natural zeolite to the Japanese quail litter on microclimate parameters, growth performance, blood gases, and other blood biochemical indicators in two Japanese quail lines. Both the zeolite-treated group and the selected line performed better than the untreated group and control line in growth performance. The microclimate characteristics were improved by adding natural zeolite in the quail litter, revealing better growth performance and health of birds. Based on this study, it can be concluded that the use of natural zeolite in the Japanese quail litter is effective in reducing the atmospheric ammonia level.

**Abstract:**

Microclimate parameters (ammonia, ambient temperature, heat index, and relative humidity) surrounding birds affect the production and health status of poultry. Therefore, this study aimed to evaluate the impact of adding natural zeolite to the litter of Japanese quail on improving microclimate parameters and its reflection in growth performance, blood gases, and blood biochemical parameters. A total of 1152 chicks were obtained from the same hatch at the 20th selection generation. Chicks were allocated into two groups based on the litter composition: Group 1: wheat straw as litter (untreated group); Group 2: 80% wheat straw + 20% zeolite (treated group). Each group consisted of 576 chicks: 410 selected line chicks and 166 control line chicks. Significant and favorable effects of the treatment on microclimate parameters during tested periods were found to favor the treated group. Either the treated group or the selected line had significantly better growth performance than the untreated group and control line. Zeolite-treated quails had significantly desirable blood gases and lower blood acidity and serum total iron binding capacity compared to the untreated group. Thus, adding natural zeolite to the litter enhanced the microclimate parameters that improved growth performance, blood gases, and blood biochemical parameters and reduced ammonia emission.

## 1. Introduction

Animal welfare scientists have emphasized the importance of controlling microclimate and air quality as relevant aspects for most categories and species of farmed animals for good housing [1]. The surrounding microclimate conditions of poultry, along with standardization, scale, and intensive husbandry processes, are essential elements influencing their growth and limiting their genetic potential [2]. Ammonia (NH_3_), ambient temperature, and relative humidity are considered among the most important microclimate parameters. Atmospheric NH_3_ is known as the major aerial pollutant in poultry barns [3], as high NH_3_ levels can severely affect broiler performance [4], increase disease risk, and jeopardize animal welfare [5]. Abnormal serum biochemical indices, hepatic damage, and reduced performance linked to oxidative stress have been found in broilers exposed to high NH_3_ concentrations [5,6,7]. Furthermore, the pressure of genetic selection for growth traits may lower the anti-stress capabilities of birds [8]. Therefore, it is crucial to improve the environmental conditions, such as temperature, humidity, harmful gases, and NH_3_ levels, in poultry houses, especially in the flocks, which are under genetic selection to obtain their best genetic potential. To decrease the adverse influence on the microclimate in the poultry house, it is required to make conditions in which the content of ammonia in the air of the poultry house is low [9]. A new approach to improve microclimate conditions is to use natural zeolite in the litter of poultry [10,11].

Natural zeolite has been used in aspects related to poultry production because of its chemical and physical properties. Zeolite is a crystalline aluminosilicate compound classified according to the framework structures’ common features. Using natural zeolite is developed by utilizing the features of gas and water absorption and ion exchange [12]. In this regard, zeolite was found to be among the most useful litter amendments due to the NH_3_ absorption, ammonium adsorption, and water retention properties of this natural mineral. In poultry, zeolite displayed affirmative results. For broiler chicks, the inclusion of zeolite as a feed supplement exhibited a favorable influence on the performance of broilers and improved their litter quality [13]. Moreover, using zeolite in the litter reduced its moisture and NH_3_ volatilization [14]. Zeolite can augment the productive performance of birds under different conditions [15,16].

It is hypothesized that the inclusion of zeolite in the litter will have a positive impact on growing quail under the genetic selection program. Thus, the purpose of this study was to evaluate the impact of adding natural zeolite to the litter of two Japanese quail lines on improving environmental conditions and its reflection in growth performance, carcass yield, blood gases, and blood biochemical parameters.

## 2. Materials and Methods

This study was conducted at the Poultry Research Center, Faculty of Agriculture, Fayoum University, Egypt, and the study protocol was approved by the Fayoum University Institutional Animal Care and Use Committee (FU-IACUC), Egypt (approval no. AEC2207).

### 2.1. Birds and Experimental Design

**Figure 1:**
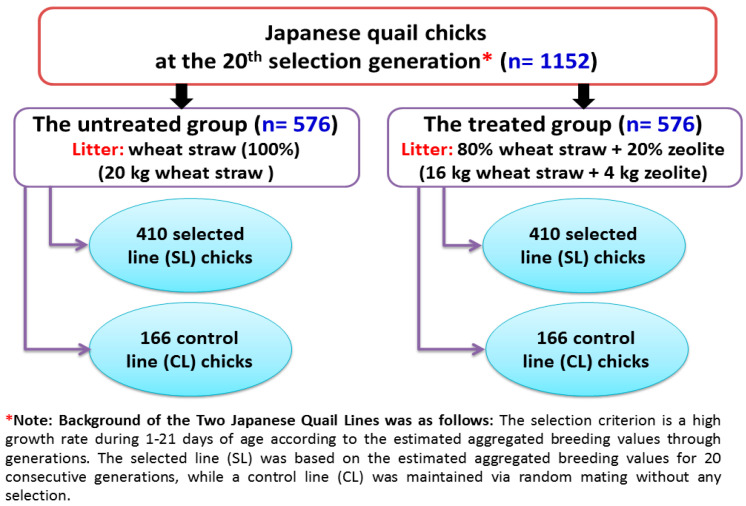
Experimental design.

A total of 1152 Japanese quail chicks (average weight ± standard deviation = 8.02 ± 0.77 g) were obtained from the same hatch at the 20th selection generation. The newly hatched chicks were wing-banded using small size plastic bands and brooded on the floor until day 42 of age. As shown in Figure 1, the birds were divided into two groups based on litter composition (5 cm deep): Group 1: wheat straw as a litter (20 kg) (the untreated group); Group 2: 80% wheat straw (16 kg) + 20% zeolite (4 kg) were properly mixed (the treated group). The wheat straw length was about 3.5–12 mm, and the moisture content was 6.0%. Zeolite granules ranged in size from 2.22 mm to 9.8 mm and were generally light-colored and hard. Each group consisted of 576 chicks: 410 selected line (SL) chicks and 166 control line (CL) chicks. Birds in the two groups were housed at a stocking density of 50 chicks/m^2^. In the same quail house, the chicks were grown in two adjacent rooms (the 1st room was treated with zeolite and the 2nd room was untreated (without zeolite)) that had the same dimensions (4 × 3 × 3 m) and orientation. Each room had two opposite windows perpendicular to the wind’s direction, providing natural ventilation. Each group consisted of selected and control chicks reared together in the same room. The birds had ad libitum access to water and feed. Quails were fed on a diet containing 24% crude protein and 2900 kcal metabolizable energy [17]. Background of the two Japanese quail lines was as follows: The selection criterion is high growth rate during 1–21 days of age according to the estimated aggregated breeding values through generations. The selected line was based on the estimated aggregated breeding values for 20 consecutive generations, while a control line was maintained via random mating without any selection.

### 2.2. Characteristics of the Product (Zeolite)

Natural zeolite (Ca, K_2_, Mg)^4^ (Al_8_ Si_40_) O_96_·24H_2_O has a cation exchange capacity of 1.50 mol/kg and it has high affinity and selectivity for NH^4+^ ions. Zeolite was purchased from A & O trading, Giza, Egypt.

### 2.3. Measurements of Microclimate

Microclimate parameters were measured once daily (during 1–2 pm) (5 measurements: 4 corners and the center of each room by one sensor for each microclimate parameter/room) when the chicks were aged from 14 to 42 days. All measurements were taken at the bird’s height (approximately 30 cm from the floor) according to Sohsuebngarm et al. [18]. Using Arduino microcontrol board ((Elecrow Company, Shenzhen, China), which has an embedded DHT11 sensor to measure ambient temperature (°C), relative humidity (%), and an MQ135 gas detector sensor for monitoring harmful gases and to detect the level of NOx, alcohol, Benzene, smoke, and CO_2_ in the poultry house (according to Orakwue et al. [19]). The heat index (HI) was mathematically calculated according to the formula cited by Wicaksono et al. [20]. The NH_3_ level was measured by an NH_3_ gas detector (Smart Sensor GM8806, BENETECH, Nanjing, China).

### 2.4. Growth Performance

Body weights at 14, 21, 28, 35, and 42 days of age (BW14, BW21, BW28, BW35, and BW42, respectively) were individually recorded to the nearest 0.01 g. The growth rate (GR) during different periods was calculated according to the following:

(1) $$Growth\;rate=\frac{[W2-W1]\times 100}{0.5[W2+W1]}$$

where W2 is the weight at the end of the period, and W1 is the weight at the beginning.

### 2.5. Carcass Traits

On day 42, forty-eight quails (6♂ SL + 6♀ SL, 6♂ CL + 6♀ CL/each treatment) were randomly taken from the two treatments for the slaughter test. The relative weight of organs (liver, gizzard, heart, and lung) was recorded as a proportion of the slaughter weight. Dressing% was calculated as the following:

(2) $$Dressing\;\%=\frac{[Carcass\;weight+Giblets\;weight]\times 100}{\left[\mathrm{Body}\mathrm{weight}\right]}$$

where Giblets (g) = the weight of three organs (gizzard, heart, and liver).

### 2.6. Blood Sampling and Laboratory Analyses

Forty-eight blood samples (6♂ SL + 6♀ SL, 6♂ CL + 6♀ CL/ each treatment) were collected from the wing vein using sterilized syringes. The blood sample/quail was collected into 2 tubes. The 1st tube contained the whole blood with an anticoagulant to determine blood gases and hematological indices (red blood cells (RBCs), white blood cells (WBCs), packed cell volume (PCV), and hemoglobin (Hb) concentration). Saturated O_2_, total CO_2_, and pH were measured in the collected whole blood using a blood gas and electrolytes analyzer (GEM PREMIER 3000, Mumbai, India). The 2nd tube (without an anticoagulant) was centrifuged (3000 rpm/10 min) to separate the serum that was saved at −20 °C until the time of laboratory analyses.

Serum total protein, albumin, urea, creatinine, NH_3_, alanine aminotransferase (ALT), aspartate aminotransferase (AST), iron, and total iron-binding capacity (TIBC) were determined using colorimetric methods and Biosystems A25 (BioSystems A.S, Barcelona, Spain). All blood biochemical indices were measured using commercial diagnosing kits (produced by Spectrum Diagnostics Company, Giza, Egypt).

### 2.7. Statistical Analyses

#### 2.7.1. Measurements of Microclimate

Data were analyzed using GLM (SAS [21]), and measurements of microclimate were subjected to a one-way analysis of variance with treatment as a fixed main effect as follows:

Y_ij_ = μ + T_i_ + e_ij_ (3)

where Y_ij_ = the observations for a trait; μ = the overall mean; T_i_ = the fixed effect of ith treatment; and e_ij_ = the random error term.

Group size: each group consisted of 576 chicks: 410 selected line (SL) chicks and 166 control line (CL) chicks.

#### 2.7.2. Growth, Blood Constituents, and Carcass Traits

The recorded data of growth, blood constituents, and carcass traits were analyzed by PROC MIXED (SAS [19]) to calculate the treatment-, line-, and sex-specific means using the following model:

Y_ijklm_ = µ + a_i_ + T_j_ + L_k_ + S_l_ + T_j_ × L_k_ + T_i_ × S_l_ + L_k_ × S_l_ + T_j_ × L_k_ × S_l_ + e_ijklm_ (4)

where Y_ijkim_: the observation for a trait, µ: the overall mean, a: the random additive genetic effect of the ith animal, T: the effect of jth treatment, L: the effect of kth line, S: the effect of lth sex, T_j_ × L_k_: the effect of the interaction of the jth treatment with the kth line, T_j_ × S_l_: the effect of the interaction of the jth treatment with the lth sex, L_k_ × S_l_: the effect of kth line with the lth sex, T_j_ × L_k_ × S_l_: the effect of the interaction of the jth treatment with the kth line with e lth sex, and e_ijklm_: is the random error term; the random variable was the birds within each line.

## 3. Results

### 3.1. Microclimate Measurements

The effects of treatment on NH_3_, harmful gases, HI, temperature, and relative humidity% during different tested periods are shown in Table 1. It was clear that there are significant and preferable effects of the treatment on NH_3_ and harmful gases during different tested periods favoring the treated group compared to the untreated group. Furthermore, HI and relative humidity% during different tested periods were lower for the treated group than the untreated group, except for the period from 21–28 days of age. Despite the increases in NH_3_ and harmful gases with increasing age, the air quality remained in favor of the treated group compared to the untreated group (Table 1).

### 3.2. Growth Performance

**Figure 2:**
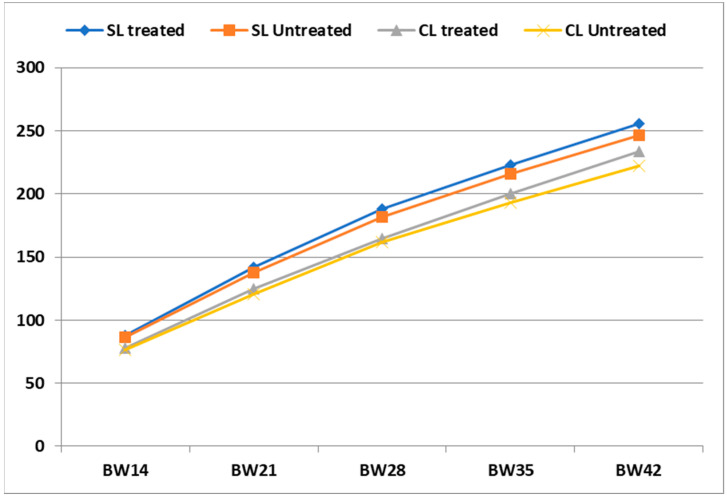
Treatment effects on body weight (BW, g) for each line separately.

The effects of treatment, line, sex, and their interactions on body weight (BW) at different ages are shown in Table 2. Quails of the SL had significantly heavier BWs than those of the CL at all ages. Except for BW14, the treated group had significantly heavier BWs compared to the untreated group, also sex significantly affected BWs in favor of females compared to males except at 14 days of age. Additionally, Figure 2 displayed that the SL and CL treated with zeolite had heavier BW than the SL and CL untreated (without zeolite). As a result, any significant differences observed between the treated and untreated groups’ performance can be attributed to the treatment itself (or addition of zeolite).

As shown in Table 3, the SL had a significantly faster growth rate (GR) than the CL during different tested periods of growth. Quails of the treated group had significantly higher GR14–21 and GR14–42 than the untreated group. Females had significantly faster GR28–35, GR35–42, and GR14–42 than males. Additionally, there were significant effects of treatment by line interaction on BW42 and GR21–28.

### 3.3. Blood Gases

Table 4 showed the effects of treatment, line, sex, and their interactions on blood gases. Zeolite-treated quails had significantly and preferably higher saturated O_2_ and lower total CO_2_ and NH_3_ compared to their counterparts in the untreated group. Regarding sex effect, males had significantly lower NH_3_ than females, whereas the line had no significant effects on blood gases. Both the saturated O_2_ and total CO_2_ showed significant impacts of the treatment by line interaction.

### 3.4. Blood Biochemical Indices

This study investigated the effect of microclimate parameters on the hematological indices, serum profile of proteins, and lipids of quails through the use of zeolite. The results in Table 5 and Table 6 show the effects of the treatment, line, sex, and their interactions on the performance of some blood biochemical parameters. Regarding the treatment effect, blood pH was significantly declined in the treated group compared with the untreated group. The SL had significantly lower blood creatinine and albumin values than the CL as affected by the line effect. Sex significantly affected both ALT and AST where males had lower estimates than females. Moreover, significant effects of treatment by line interaction appeared on ALT, creatinine, urea, and albumin.

### 3.5. Iron Profile and Haematological Parameters

Significantly lower serum iron levels and a rise in TIBC levels were seen in the untreated group than in the treated group. However, according to the statistical analysis, there were no significant differences in Hb, PCV, RBCs, and WBCs between the two groups. The line significantly affected PCV with a higher value for the CL than the SL. Males had significantly higher Hb and PCV than females as shown in Table 6.

### 3.6. Carcass Traits

The performance of some carcass traits as affected by treatment, line, sex, and their interactions is presented in Table 7. Treatment had a significant effect on gizzard % favoring treated quails compared to those from the untreated group. Additionally, the previous results refer to numerically and preferably higher dressing % for the treated than the untreated groups. However, the line significantly affected the dressing % and gizzard %, favoring the SL compared to the CL. Females had significantly higher liver % than males. Effects of significant treatment by line interaction were found for liver %.

## 4. Discussion

Poultry house air quality contributes to sustainability due to its influence on the health of birds and humans working there, and as a factor affecting the environment directly [22]. Thus, controlling the microclimate conditions is one of the innovative solutions in public health and environmental protection necessary to encourage the development of modern poultry farming. Measurements of the microclimate during different tested periods were significantly influenced by the addition of zeolite to the litter. This may be due to the fact that zeolite has large porosity and surface area, which have a beneficial effect on the absorption of liquids, such as water, NH_3_, organic liquids, and gases, such as volatile organic compounds and hydrocarbons [23]. Additionally, Schneider et al. [14] stated that zeolite can easily absorb toxic gases from the air, such as ammonia. Finally, the current study developed a new litter amendment composed of 80% wheat straw plus 20% zeolite which may be used as an effective solution for reducing NH_3_ emissions from the quail litter and improving microclimate conditions. Moreover, zeolites have unique chemical and physical characteristics that can be used as a part of the litter in poultry production to mitigate the pollution. Thus, improved microclimate conditions can boost bird comfort and reduce the hazard of ammonia toxicity in the birds’ house [24], leading to the improvement of productive performance. This is of great importance for the biosafety and hygiene of production.

In the current study, the growth performance of the treated group was significantly enhanced over the untreated group by the addition of zeolite to the litter. Zhu et al. [25] and Wei et al. [26] reported similar trends in ducks and chickens. Basha et al. [27] mentioned that the natural zeolite addition in the litter in the broiler house increased the productive performance of chicks. This may be attributed to higher hematic NH_3_ levels, which can accelerate the detoxification of NH_3_ in the muscle, brain, and liver, which is a very energy-intensive process [26], which translates into a reduction in the energy required for growth and production, which has an adverse impact on growth performance. Zeolite treatment of the litter resulted in significant differences between the treatment averages in final body weight and average daily weight [11]. Similarly, Eleroğlu and Yalçın [28] revealed statistically significant differences in broiler live weight, which were consistent with the findings of the current study. This may be due to the structure of zeolite allowing for ion exchange, molecular “sieving,” absorption, dehydration, diffusion, catalysis, and reversible dehydration, which improve microclimate conditions surrounding the quails. The litter treatment using zeolite as a management practice to boost broiler performance and reduce ammonia emission was able to improve the performance of birds by having a negligible impact on the main odor-producing culprits and microbial activities in the litter [11]. In contrast, Altan et al. [29] used zeolite to treat litter and reported insignificant differences in the live BW gain of birds among the treatments.

The effects of sex and selection on the growth performance parameters were examined in the current study, the literature revealed differences between lines of various genetic backgrounds. The selection is a successful approach for quail performance and genetic enhancement, which may result in some alterations of physiological and metabolic processes that affect characteristics related to growth or egg production [30,31,32,33]. Since the growth trends of the sexes varied, females had significantly better BW and GR than males. According to the results of Narinc et al. [34] and Elkomy et al. [35], females were consistently heavier than males for BW at various ages. Furthermore, there was a noticeable sexual dimorphism in Japanese quail BW, favoring females over male counterparts as a result of male sexual activity caused by hormonal alterations. Furthermore, Taskin et al. [36] found that across selection generations, sex was a substantial source of variance for BW at all ages. On the contrary, Mahmoud et al. [37] found an insignificant sex effect on BW at all ages. In addition, treated quails showed significantly faster GR14–21 and GR14–42 than the untreated group; Zhu et al. [25] and Wei et al. [26] reported similar trends in ducks and chickens.

Improved microclimate conditions can boost the blood physiology of birds. Determining numerous blood physiology parameters such as blood gas concentration and blood biochemical indices may indicate the suitability of housing for birds. The zeolite-treated group had significantly better blood saturation levels of O_2_, CO_2_, and NH_3_ when compared to the untreated group. These results are in harmony with the findings obtained by Wei et al. [38,39] and Zhu et al. [25], who indicated that exposure to high NH_3_ levels had a significant impact on plasma NH_3_ levels in chickens and ducks. Zeolite can actively adsorb carbon dioxide, ammonia, mercaptans, and hydrogen sulfide, remove toxins, and improve immunological responses [15], thus the inclusion of zeolite in the litter can decrease blood gases (saturated oxygen, total carbon dioxide, and ammonia).

The inclusion of zeolite in the litter can enhance the chemical, microbiological, and physical integrity of the litter, and consequently, can boost the performance, hygiene, and ambience of poultry [40], improving the physiological status of birds. The recent findings may validate that zeolite litter treatment decreased the health risks for quails, which was reflected in some blood biochemical traits. The blood pH of the treated group significantly declined compared to the untreated group. A similar trend that blood pH was higher for the untreated group than the treated group was reported by Borges et al. [41] and Wasti et al. [42], indicating the occurrence of slight alkalosis which may have resulted from the higher panting rate in birds of the untreated group during challenging higher heat stress. The current study indicated that the serum concentrations of AST, ALT, blood urea, creatinine, and protein profile did not differ significantly between the two treatment groups; this may be due to the fact that atmospheric NH_3_ did not exceed the harmful level in the two treatment groups which contradicted those results of the previous studies reported by Zhu et al. [25], Chen et al. [43], and Lu et al. [44]. Furthermore, Zhang et al. [7] reported that birds exposed for an extended period to high concentrations of atmospheric NH_3_ may have chronic liver and renal damage. Blood gases and serum biochemical examinations indicate the affirmative influence of the addition of zeolite in the litter of Japanese quail. Currently, adding zeolite to the litter for the genetically selected quail based on the growth rate could be proposed. However, it is equally significant to continue this kind of research due to its scarcity in science. There was evidence of significant sex-related differences in ALT and AST estimates, demonstrating that females had higher values of AST and ALT than males, which may be explained by the physiological changes in metabolism in female birds due to maturation and attaining egg laying [45]. Similar findings were reported by Udoh et al. [46]; however, Scholtz et al. [47] reported significant differences in ALT and AST with males exhibiting lower estimates than females.

Iron is a necessary constituent of Hb, which is the O_2_-carrying protein in RBCs [48]. After improving microclimate conditions, serum iron concentration was significantly increased in birds treated with zeolite compared with the untreated birds. The untreated group has shown a significant decrease in serum iron associated with the increase in TIBC level, indicating that birds of this group were experiencing some stressful conditions such as psychological stress which has been shown to activate the hypothalamic–pituitary–adrenal axis system, which resulted in elevated levels of adrenocorticotropic hormone in the blood and decreased levels of serum iron, hepatic iron enrichment, and the enrichment of iron overload [49,50]. This study found increases in Hb, PCV, and RBCs together with decreased blood oxygen and increased carbon dioxide saturation in the untreated group, which may be associated with the increased metabolic activity required to fulfil the energy requirements for growth, especially when birds are kept under challenging inhalation conditions, including higher atmospheric NH_3_ and harmful gases levels along with higher HI measurements. Similar findings were reported by Olanrewaju et al. [51] and Asif et al. [52]. The enhancement in the hematological parameters in the zeolite-treated birds can be attributed to the role of zeolite in improving air quality in the birds’ housing. There is no available literature on using zeolite in the litter for quails and its influence on serum iron, TIBC, and blood hematology; consequently, further investigations on this point are needed.

The current experiment indicated that carcass traits were decreased in the untreated group, which may be associated with suffering from some stressful conditions. This is in agreement with some previous studies [5,53]. The current results indicated that improving microclimate conditions during the growth period non-significantly boosted the dressing % at the marketing age of Japanese quail. The present study indicated that the inclusion of zeolite in the litter resulted in a favorable impact on gizzard, possibly due to the effect of the birds in this group eating the zeolite in the litter. Similarly, the addition of zeolite to the diet significantly increased the relative weight of edible organs including the gizzard [54]. In broiler chickens, Banaszak et al. [55] indicated a significantly higher body weight and carcass weight in the treated group with aluminosilicates in feed or litter compared to the control group. Furthermore, the aluminosilicates-treated group had significantly high weights of wings and neck with the skin but did not affect the dressing percentage. Therefore, future research should deeply address how the zeolite group showed better growth performance but not a significant increase in the dressing percentage.

The present results showed that the genotype of a bird and the microclimate surrounding the quails had a substantial impact on a bird’s physiological and productive performance. The results also showed a significant effect of line-by-treatment interaction for some studied traits (BW42, GR21–28, saturated O_2_, total CO_2_, ALT, creatinine, urea albumin, and liver %). Similarly, Erdem et al. [56] indicated that young layer chickens’ genotype and dietary environment influenced their disease resistance. Interaction between the genetics and environment might decrease the effectiveness of breeding programs [57]. Because of this, genotype by environment interaction is important for breeding programs’ effectiveness and sustainability.

Finally, microclimatic measurements are necessary constituents for boosting the healthy environment for poultry. The current results suggest that the birds of the selected line were likely trying to take advantage of the improved microclimate conditions. The presented findings provide a tentative signal that zeolite influences ammonia release in quail houses, decreasing the adverse health significances of ammonia emissions. In our opinion, this solution, adding natural zeolite to the litter (ratio: 20% zeolite: 80% wheat straw), to improve microclimate conditions and reduce the ammonia emissions from the litter, can be considered promising in poultry farms. The present study suggested that improvement of microclimate conditions by adding zeolite to the litter of genetically selected quails for fast growth rate could be a promising area for future research.

## 5. Conclusions

Using natural zeolite as 20% of the quail wheat straw litter is considered a promising solution for the surrounding problems related to the microclimate conditions of poultry, since it led to an enhancement of the quails’ health, physiological status, growth performance, and microclimate parameters, which was accompanied by a decrease in the ammonia emission.

## Figures and Tables

**Table 1:** Effect of treatment with zeolite on microclimate measurements during different tested periods.

Treatment	Tested Period (Days)	Ammonia (ppm)	Harmful Gases (ppm)	Heat Index (°C)	Temperature (°C)	Relative Humidity (%)
Treated (zeolite)	14–21	5.67	22.97	34.40	33.4	44.00
Untreated (without zeolite)		9.31	40.89	36.19	33.43	46.41
SEM		0.96	3.57	1.19	0.33	0.80
Probability		0.0078	0.0008	0.0063	0.6184	0.0469
Treated (zeolite)	22–28	5.67	28.06	38.67	35.30	42.64
Untreated (without zeolite)		16.38	45.75	40.15	35.94	43.54
SEM		1.20	4.08	0.55	0.29	0.75
Probability		0.0001	0.0035	0.0580	0.1238	0.4047
Treated (zeolite)	29–35	6.49	28.87	35.80	33.41	45.64
Untreated (without zeolite)		16.53	48.67	39.36	34.66	49.03
SEM		1.34	3.66	0.74	0.37	0.53
Probability		0.0014	0.0006	0.0015	0.2081	0.0001
Treated (zeolite)	36–42	9.71	49.33	33.61	32.80	42.86
Untreated (without zeolite)		18.07	80.53	36.44	33.72	49.04
SEM		1.36	7.49	0.53	0.28	1.65
Probability		0.0001	0.0059	0.0006	0.3170	0.0001

**Table 2:** Least square means for the body weight (BW, g) at different ages studied as affected by treatment, line, and sex effects.

Items	BW14	BW21	BW28	BW35	BW42
Treated (zeolite)	82.24	133.57	179.80	219.81	247.07
Untreated	82.15	127.19	170.07	206.21	231.09
SEM	1.27	0.97	0.72	1.19	1.47
Selected line	86.22	141.49	180.20	220.88	268.19
Control line	77.17	123.47	155.66	190.14	228.97
SEM	0.91	0.97	1.14	1.17	1.43
Females	83.25	133.53	176.75	213.93	249.43
Males	81.14	129.15	171.11	203.09	229.73
SEM	0.94	1.01	1.19	1.2	1.48
Probability					
Treatment	0.9436	0.0018	0.0260	0.0001	0.0001
Line	0.0001	0.0001	0.0001	0.0001	0.0001
Sex	0.1131	0.0021	0.0008	0.0001	0.0001
Treatment ∗ line	0.7413	0.2206	0.7463	0.1909	0.0442
Treatment ∗ sex	0.6361	0.5787	0.8132	0.7411	0.3282
Line ∗ sex	0.9297	0.9281	0.9968	0.2422	0.2697
Treatment ∗ line ∗ sex	0.9903	0.4482	0.5638	0.8908	0.3129

**Table 3:** Least square means for the growth rate (GR, %) during different periods studied as affected by treatment, line, and sex effects.

Items	GR14–21	GR21–28	GR28–35	GR35–42	GR14–42
Treated (zeolite)	47.57	29.51	20.02	11.68	100.11
Untreated	43.03	28.85	19.21	11.38	95.10
SEM	0.78	0.46	0.45	0.37	0.84
Selected line	48.54	24.07	20.29	19.35	102.69
Control line	46.15	23.06	19.94	18.53	99.17
SEM	0.94	0.45	0.44	0.35	0.98
Females	46.39	27.86	19.03	15.32	99.90
Males	45.66	27.95	17.09	12.31	95.60
SEM	0.81	0.46	0.45	0.36	0.86
Probability					
Treatment	0.0001	0.2024	0.1544	0.3024	0.0126
Line	0.0420	0.0057	0.0052	0.0183	0.0036
Sex	0.9781	0.7741	0.0182	0.0001	0.0117
Treatment ∗ line	0.3761	0.0460	0.1195	0.7067	0.3387
Treatment ∗ sex	0.5552	0.3608	0.9478	0.5709	0.2964
Line ∗ sex	0.8108	0.5241	0.1433	0.5069	0.7544
Treatment ∗ line ∗ sex	0.9471	0.0151	0.2635	0.1500	0.7497

**Table 4:** Least square means for blood gases as affected by treatment, line, and sex effects.

Items	Saturated Oxygen (%)	Total Carbon Dioxide (mmol/L)	Ammonia (µmol/L)
Treated (zeolite)	96.00	22.52	217.21
Untreated	93.38	27.43	343.58
SEM	0.50	0.50	23.72
Selected line	94.88	25.19	249.92
Control line	94.50	24.76	310.88
SEM	0.50	0.50	24.81
Females	94.00	24.77	330.00
Males	95.38	25.18	230.79
SEM	0.48	0.48	24.59
Probability			
Treatment	0.0011	0.0001	0.0014
Line	0.5982	0.5464	0.0952
Sex	0.0627	0.5664	0.0093
Treatment ∗ line	0.0302	0.0001	0.7971
Treatment ∗ sex	0.5982	0.0650	0.4767
Line ∗ sex	0.3824	0.0931	0.0644
Treatment ∗ line ∗ sex	0.5982	0.5487	0.0124

**Table 5:** Least square means for blood biochemical parameters as affected by treatment, line, and sex effects.

Items	pH	ALT (U/L)	AST (U/L)	Urea (mg/dL)	Creatinine (mg/dL)	Total Protein (g/dL)	Albumin (g/dL)	Globulin (g/dL)
Treated (zeolite)	7.23	17.00	179.88	6.71	0.50	4.83	1.79	3.04
Untreated	7.38	15.29	143.00	6.88	0.46	5.04	1.85	3.19
SEM	0.03	0.99	14.96	0.39	0.03	0.15	0.03	0.13
Selected line	7.32	16.92	152.38	6.58	0.44	4.97	1.74	3.23
Control line	7.29	15.38	170.50	7.00	0.52	4.90	1.91	3.00
SEM	0.03	1.01	14.95	0.38	0.04	0.15	0.03	0.13
Females	7.30	20.13	190.13	6.75	0.45	4.75	1.81	2.94
Males	7.31	12.17	132.75	6.83	0.50	5.12	1.84	3.28
SEM	0.02	1.08	14.74	0.40	0.02	0.14	0.03	0.14
Probability								
Treatment	0.0001	0.2369	0.0941	0.7587	0.0908	0.3367	0.1189	0.4405
Line	0.2080	0.2845	0.4000	0.4448	0.0084	0.7565	0.0001	0.2422
Sex	0.4887	0.0001	0.0122	0.8778	0.2334	0.0973	0.3603	0.0963
Treatment ∗ line	0.0537	0.0042	0.9023	0.0048	0.0002	0.4635	0.0002	0.9949
Treatment ∗ sex	0.3642	0.1771	0.1886	0.0397	0.0020	0.5114	0.0017	0.1801
Line ∗ sex	0.2629	0.0590	0.4195	0.8778	0.0001	0.2217	0.4504	0.1407
Treatment ∗ line ∗ sex	0.0340	0.5792	0.2465	0.5401	0.0150	0.3761	0.6832	0.2955

AST: aspartate aminotransferase; ALT: alanine aminotransferase.

**Table 6:** Least square means for iron profile and hematological parameters as affected by treatment, line, and sex effects.

Items	Iron (µg/dL)	TIBC (µg/dL)	Hb (g/dL)	RBCs (10^6^/mm^3^)	PCV (%)	WBCs (10^3^/mm^3^)
Treated (zeolite)	302.55	429.36	12.52	3.55	41.43	26.60
Untreated	255.96	493.34	12.87	3.56	43.75	27.28
SEM	19.57	17.1	0.46	0.06	1.51	1.60
Selected line	296.80	452.56	12.23	3.52	40.20	25.00
Control line	261.71	470.14	13.16	3.59	44.99	28.88
SEM	18.54	17.38	0.47	0.08	1.50	1.61
Females	278.90	458.24	11.14	3.59	37.06	26.44
Males	279.62	464.46	14.25	3.51	48.12	27.44
SEM	19.2	17.66	0.43	0.07	1.21	1.59
Probability						
Treatment	0.0492	0.0172	0.5944	0.8518	0.2842	0.7686
Line	0.2088	0.4886	0.1660	0.5064	0.0328	0.1019
Sex	0.9792	0.8054	0.0001	0.3964	0.0001	0.6637
Treatment ∗ line	0.0574	0.0548	0.3473	0.0832	0.1101	0.2651
Treatment ∗ sex	0.0751	0.0037	0.5274	0.2265	0.1567	0.1635
Line ∗ sex	0.0147	0.0131	0.2141	0.0086	0.7028	0.2835
Treatment ∗ line ∗ sex	0.0464	0.2234	0.4573	0.0411	0.7318	0.0682

TIBC: total iron-binding capacity; Hb: hemoglobin; RBCs: red blood cells; PCV: packed cell volume; WBCs: white blood cells.

**Table 7:** Least square means for dressing % and relative organs as affected by treatment, line, and sex effects.

Items	Dressing %	Heart %	Lung %	Liver %	Gizzard %
Treated (zeolite)	74.49	0.89	0.69	2.01	1.93
Untreated	72.20	0.84	0.70	1.92	1.75
SEM	2.39	0.05	0.03	0.07	0.05
Selected line	76.57	0.82	0.71	1.92	1.76
Control line	73.05	0.91	0.72	2.05	1.93
SEM	2.98	0.04	0.03	0.06	0.05
Females	74.33	0.81	0.69	2.10	1.88
Males	72.15	0.92	0.71	1.83	1.81
SEM	2.38	0.03	0.02	0.06	0.06
Probability					
Treatment	0.5002	0.3618	0.8006	0.3507	0.0156
Line	0.0410	0.0930	0.7502	0.3437	0.0261
Sex	0.9678	0.0549	0.5307	0.0055	0.3181
Treatment ∗ line	0.5402	0.0831	0.2073	0.0029	0.9359
Treatment ∗ sex	0.6002	0.0204	0.0220	0.7421	0.9488
Line ∗ sex	0.6986	0.3904	0.2346	0.0105	0.0230
Treatment ∗ line ∗ sex	0.5702	0.2864	0.7803	0.0676	0.5264

## Data Availability

The data presented in this study are available on request from the corresponding author.
